# Education for automation—reaching the right people?

**DOI:** 10.1155/S1463924679000699

**Published:** 1979

**Authors:** Clarence H. Annett

**Affiliations:** The University of Kansas Lawrence Kansas 66045 USA


Now more than ever instrument companies woo potential customers with the claim that their instruments are the panacea for all analytical problems. Often this claim is associated with the introduction of microprocessor technology. The unsuspecting analyst may find that he has purchased an instrument which is more sophisticated than he needs; he cannot be expected to predict that the instrument will meet any future requirements. If he has not been fully involved in all stages of purchase he may find himself with an instrument which is incapable of the requirements to be placed upon it.

It cannot be stressed too often that an essential requirement is a full and detailed specification of the analytical needs. The specification should be assessed by the analyst, and systems designer/manufacturer in cooperation. A solution meeting this specification will then be designed which makes use of all the available resources including in-house and commercial technical and economic considerations. Only in this way can new technology be effectively and economically introduced.

The analyst has an important role in the implementation of automation. It is clearly not a simple role. It requires a commitment to the overall objectives of automation and the involvement and encouragement by managment. A willingness to transpose ideas across disciplinary boundaries is an essential requirement for all concerned. The most difficult constraint to overcome in the introduction of successful automation is a proper understanding of the chemistry involved and correct use of materials of construction.

This journal provides a medium for the discussion of automation problems and articles in it will hopefully overcome the barriers to automation. Recently the symposium 'Analysis 1979' brought together clinical and industrial chemists to discuss papers of mutual interest. It formed a valuable exchange of ideas and philosphies and it is hoped that future meetings will be organised along similar lines. The papers presented at this meeting had considerable merit and for future occasions it is hoped that a larger audience can be attracted.

*Peter B. Stockwell*

### Education for automation–reaching the right people?

In the April issue of this journal, Professor Howard V. Malmstadt presented a commentary on the problem of education in automated analysis. As an analyst who was trained in the classical methods and had to learn automation techniques by laboriously extracting material from a variety of journals and other sources, I have no quarrel with his contention that an integrated program of education is sorely needed in the training of automatic analysis as part of advanced degree programs. However, I believe that it overlooks a crucial but parallel point: the acceptance of automation for routine laboratory work will not ultimately depend on these people, but instead on others whose scientific training is considerably less than the Ph.D. In the specific case I wish to discuss, the hospital/clinical setting, the people having the most influence on automation decisions will belong to one of two groups, administrators and laboratory technicians. To my knowledge, no training programs appropriate for either group exist anywhere.

The small doctor's office or clinic is not important here because the number of blood, urine, and other samples processed is small enough to be conveniently handled by non-automated techniques. The large hospital, however, is a different story. A typical 500 bed hospital will process upwards of 20,000 blood samples each year, and the number of urine samples will be similar. Clearly, automated analysis techniques are suggested in order to handle the sheer volume of samples, yet few hospitals have anything more automated than a sample changer in their laboratory. When older equipment wears out, it is replaced by similar non-automated instruments rather than by more modern automated ones. The laboratory is thus crowded with technicians, who must work feverishly to keep up with the work load. The failure of any instrument is a disaster, as there are seldom spares, and upon repair many hours of overtime are required for the technicians to catch up. If for any reason the work load increases, only one solution is considered – hire more technicians.

This situation is perpetuated by the hospital administration, whether it be the medical personnel or the business personnel. First, the cost vs. benefit of automated analysis has never been explained to them. They see high price tags on automated instruments but do not realize what beneficial change the instruments would effect on the clinical lab. University-style classes will not help these people, as most have neither the advanced technical background nor the time required to digest such a course. Short courses, seminars, and continuing education classes are desperately needed to fill this gap. Such courses presently do not exist, nor does literature at a sufficiently non-technical level that a course could be built around it. Courses of this type would also help alleviate a second problem, namely that many administrators still consider microprocessors and automated instruments as "big boy's toys" rather than practical devices and are thus reluctant to commit money for them. This problem is aggravated by the mystic vocabulary which surrounds computer devices in general.

Occasionally a hospital will be blessed with a far-sighted or well-educated administrator who can look beyond these difficulties and raise a new point: few, if any, training programs for clinical laboratory technicians include automated analysis. Thus if the hospital buys automated instruments, the technicians will not be able to operate them, and educational opportunities for them to learn how are almost nonexistent. For this same reason, the technicians themselves seldom support a change to automation even though it would make their job easier and more efficient.

Thus although the number of samples and the number of tests per sample make the hospital laboratory a logical candidate for automation, few hospitals have accepted it because it has neither support from the administrators who must pay for it nor from the technicians who would use it. This lack of support arises from a lack of education as to what automation can do.

There is no situation in industry that corresponds to this. The myriad of government agencies and regulations which affect product quality, air and water pollution, and worker health and safety have forced industrial laboratories to undertake substantial testing programs. Automation has become both the accepted and the preferred method for conducting the requisite number and type of tests to ensure compliance. The people responsible for such testing programs are usually those to whom the usual automation class is addressed, Ph.D's or others with advanced scientific training.

*Continued on page 243*

In summary, it does not make sense to train scientists in automation without also educating the non-scientists who will be the decision makers. Only such education, which is not now available, will speed the final transition of automation from a laboratory curiosity to a working laboratory tool.

*Clarence H. Annett*
*The University of Kansas*
*Lawrence, Kansas 66045*
*U.S.A.*

*Following is a reply to Annett from D.S. Young*

The clinical laboratory director abrogates one of his responsibilities if he allows a hospital administrator to dictate the choice of instrumentation regardless of whether it is automated or not. The administrator is untrained in any facet of laboratory work and should not have to be concerned with selection of equipment. The solution to the problem of proper choice of instrumentation raised by Annett is not education of administrators in analytical techniques and automation but education of clinical chemists and laboratory directors in business management practices. Whether one likes it or not clinical laboratories and hospitals have to be run as businesses with decisions made not only on the basis of quality of service but also on their cost-effectiveness. Thus, any new instrumentation should be evaluated in terms of the quality of results produced and the cost per test performed. In presenting a case for new equipment the laboratory director should demonstrate the improved cost-effectiveness of the new instrument. The hospital administrator is more concerned with this than other factors and his final endorsement of the purchase will, to a large extent, be dictated by the reduction in costs that will be achieved by the new equipment. While many clinical chemists and clinical pathologists have not had proper training in workload recording and cost-accounting, most training programs in clinical chemistry and many medical residency programs do include such training. There are also several books that deal with clinical laboratory management and include discussions of cost-accounting. Laboratory directors should expect to have to justify recommended purchases and the justifications should be in a form that is readily understood by the individual who has the final say.

If a technician is trained in the typical hospital laboratory he will almost certainly be exposed to some type of automation as few hospital laboratories associated with technician or technologist training do not have some automated equipment. There is of course a large variety of automated instruments and it is impossible even in the largest center for an individual to have experience with every type of automated instrument. Individuals entering the field of medical technology recognize the strong influence of instrumentation in the field. Some are deterred by it and tend to gravitate away from clinical chemistry towards microbiology, blood banking and hematology even though automation has made considerable strides in the area of hematology. Those who enjoy working with machines have found no problems in most instances in adapting themselves from a manual instrument to a mechanized way of doing the same thing. Obviously there is an onus on a laboratory director to discuss with his staff the impact of introducing automation in a previously non-automated laboratory and to ensure that the staff is properly trained, if they have not previously used the same equipment. He has the same responsibility even if the equipment is not automated.

In summary, I do not believe that the problems discussed by Annett are real if laboratory directors are properly trained. If there is a need for action, it is in better training of laboratory directors.

*D.S. Young*
*Mayo Clinic,*
*Rochester,*
*Minnesota,*
*U.S.A.*

### Notes for Contributors

### Presentation of manuscripts

Manuscripts should be typed (double-spaced) on one side of the paper only and with generous margins. The title should be brief and informative avoiding the word "new" and its synonyms. The full list of authors with their affiliations and full address(es) should appear on the title page. On a separate sheet an abstract of no more than 150 words is required. This should succinctly describe the scope of the contribution and highlight significant findings or innovations. It should be written in a style which can easily be translated into French and German.

*The Concise Oxford Dictionary* and Fowler's *Modern English Usage* (both published by Oxford University Press) should be used as the standard for spelling and grammar. Abbreviations should be limited to those generally recognised, or where a frequently occuring term is abbreviated it should, in the first instance, be explained thus "flow injection analysis (FIA) ..." and the abbreviation used thereafter. Abbreviations, for standard measures and units should follow SI recommendations. There are various publications giving guidance on the use of SI units.

References should be indicated in the text by numerals following the author's name, i.e. Skeggs [6]. On a separate sheet of paper, list all references in numerical order thus:

[6] Skeggs, L.T., *American Journal of Clinical Pathology,* 1959, **28**, 311.

Note that journal titles are given in full. Where there is more than one author, the form Foreman et al. should be used in the text but all authors should be named in the list of references. When reference is made to a chapter in a book the reference should take the following form:

[7] Malmstadt, H.V. in "Topics in Automatic Chemistry" Ed. Stockwell P.B. and Foreman J.K. 1978 Horwood, Chichester, pp. 68-70.

Only work which has been published or has been accepted for publication should be cited. Avoid the citation of documents which are subject to restricted circulation, patent literature, unpublished work and personal communications. The latter can be mentioned in the text in parenthesis.

To illustrate a paper line diagrams are preferred to photographs. Photographs should only be used when they significantly add to the discussion. Diagrams, charts and graphs should be carefully drawn in black ink on stout card or heavy quality tracing paper. Most illustrations are reduced for publication; to allow for this originals should be between 16 and 36 cm wide (the depth must not exceed 50 cm). The lettering of diagrams should be sufficiently clear to withstand reduction. Except in the case of proper names, all lettering should be in lower case print. If photographs are used they must be supplied in the form of clear, unmounted, glossy, black and white prints. "Instant" photographs are not normally acceptable. All illustrations must be identified on the reverse showing the figure number and the author's name.

Each illustration should have a fully explanitory caption. Captions should be typed together on a separate sheet of paper; they must *not* be an inseparable part of the illustration.

